# Mortality of Inshore Marine Mammals in Eastern Australia Is Predicted by Freshwater Discharge and Air Temperature

**DOI:** 10.1371/journal.pone.0094849

**Published:** 2014-04-16

**Authors:** Justin J. Meager, Colin Limpus

**Affiliations:** Threatened Species Unit, Department of Environment and Heritage Protection, Dutton Park, Queensland, Australia; Ecole Normale Supérieure de Lyon, France

## Abstract

Understanding environmental and climatic drivers of natural mortality of marine mammals is critical for managing populations effectively and for predicting responses to climate change. Here we use a 17-year dataset to demonstrate a clear relationship between environmental forcing and natural mortality of inshore marine mammals across a subtropical-tropical coastline spanning a latitudinal gradient of 13° (>2000 km of coastline). Peak mortality of inshore dolphins and dugongs followed sustained periods of elevated freshwater discharge (9 months) and low air temperature (3 months). At a regional scale, these results translated into a strong relationship between annual mortality and an index of El Niño-Southern Oscillation. The number of cyclones crossing the coastline had a comparatively weak effect on inshore marine mammal mortality, and only in the tropics. Natural mortality of offshore/migratory cetaceans was not predicted by freshwater discharge, but was related to lagged air temperature. These results represent the first quantitative link between environmental forcing and marine mammal mortality in the tropics, and form the basis of a predictive tool for managers to prepare responses to periods of elevated marine mammal mortality.

## Introduction

Many marine mammal species are conservation dependent, with low population growth rate and poor resilience to human impacts [1], [2]. Managing anthropogenic mortality of marine mammals with tools such as precautionary reference points [3], [4] depends on an understanding of the dynamics of natural mortality. Natural mortality is rarely constant in space and time-even for large, long-lived mammals [1], [5]. The risk of disease, parasitism, starvation and predation varies with seasonal fluctuations and natural perturbations such as climatic phenomena and habitat disturbance [2], [6]–[8]. Understanding the relationship between extrinsic environmental forcing and mortality in marine mammal populations is an important first step in predicting possible impacts of extreme weather events and climate change [7].

Australia is recognised as both a hotspot for marine mammal diversity [9] and as a region that experiences extreme climatic and rainfall variation [10]. Tropical and subtropical eastern Australia supports a globally significant population of the herbivorous dugong, *Dugong dugon* [2], and a diverse assemblage of resident, transient and migratory cetaceans [11], [12]. Two species of tropical dolphins of special conservation concern are resident in the region, at least one of which is endemic to Australasia [13], [14]. Climatic variation in eastern Australia is largely dominated by the El Niño-Southern Oscillation phenomenon [15], [16], and often alternates between drought- and flood-dominated periods. Over the long term (i.e. since the early 1900s), air temperature and sea surface temperature (SST) in the region have significantly warmed and are predicted to continue to increase [17]. Exactly how warming temperatures will interact with ENSO is uncertain, but precipitation extremes and the frequency of severe weather events such as floods, storms and cyclones are predicted to increase into the future [17]. Such extreme weather events were exemplified by summer of 2010-11, when high sea-surface temperatures and a strong La Niña resulted in widespread and severe flooding in eastern Australia. A very large tropical cyclone then crossed the coast in northern Queensland (Cyclone Yasi) in February 2011. Several months later, high numbers of stranded and dead dugongs and dolphins were reported along the Queensland coast [18], [19].

Episodic floods and cyclones have long been suspected to be key drivers of dugong mortality [20]–[22], but statistical evidence or predictive models have not been forthcoming because long-term data over a relevant spatial scale have not until recently been available. It is thought that the primary reason for elevated dugong mortalities following severe weather events is because of large-scale disturbance to seagrass pastures [20]–[22]. The effect of floods and cyclones on other marine mammals, such as inshore dolphins, has received much less attention, but impacts on prey resources for inshore dolphins are also well documented (i.e. fish, cephalopods and crustaceans: [23], [24]). Further, the impacts of freshwater discharge on estuarine and coastal ecosystems are not limited to periods of flood; sustained periods of high or low freshwater discharge have a strong influence on seagrass pastures and prey assemblages [23], [25].

Impacts of freshwater discharge and cyclones are likely to be most pronounced in shallow, coastal or estuarine-influenced areas that represent critical habitat for dugongs and inshore dolphins [26], [27]. Conversely, the effects of freshwater discharge and cyclones should be less pronounced on species that do not feed in coastal waters or that are present in coastal waters for short time periods, such as offshore, transient or migratory cetacean species.

In the current paper, we test the prediction that freshwater discharge and cyclones have a strong impact on mortality of dugongs and resident inshore dolphins, but not on mortality of offshore/migratory cetaceans, by developing nonparametric models to empirically explore a 17-year dataset of marine mammal mortality spanning a latitudinal gradient of 13° (>2000 km of coastline). Because marine mammals are affected by both past and present conditions, we also examine how the timing and duration of environmental forcing influences marine mammal mortality.

## Methods

### Biological data

The Queensland Marine Wildlife Strandings and Mortality Program maintains records of sick, injured, incapacitated or dead marine wildlife in Queensland, Australia, in an online Oracle database (StrandNet). Records are obtained from government departments, community groups, businesses, environmental organisations, and the general public via a telephone hotline. Records lodged in StrandNet include information such as location, date, sex, life-history stage and carcass condition. All records are verified by experts. The probable cause of death is established through necropsies by veterinarians, examination of carcasses by trained staff or, in some cases, through photos and/or case histories. Where there is no evidence for a probable cause of the death, it is recorded as ‘unknown’.

Marine mammal strandings and mortalities have been systematically recorded along the ‘urban’ coast of Queensland (Cairns to the Queensland-New South Wales border) since 1996. Within this area, coverage is most comprehensive in densely populated and highly trafficked regions, such as southern Queensland, and in areas regularly patrolled by marine park rangers. However, marine mammal strandings attract significant attention from the public and there are very few locations along the Queensland coast where beach-washed marine mammals are not reported. In the current study, we analyse relative spatio-temporal patterns of natural marine mammal mortality (i.e. by month and latitudinal block) rather than estimating true natural mortality, because the proportion of marine mammal carcasses which reach the shore is unknown (*sensu* [28]).

Data were exported from StrandNet on 21/08/2013 and were summed over a 1° gradient from the Queensland-New South Wales border (28°S) to northern Queensland (16°S) for each month from January 1996 to December 2012 (i.e. 13 latitudinal blocks). These data spanned in excess of 2000 km of coastline that included a range of marine systems, such as open-ocean beaches, protected bays, estuaries and coral reefs. Only cetacean and dugong mortalities that were either attributed to natural or unidentified causes are examined in this paper. No mass mortality events (e.g. mass strandings) occurred in the dataset.

### Environmental predictors

Two climatic indicators: the Southern Oscillation Index (SOI, based on the difference in sea level pressures between Tahiti and Darwin, Australia); and SST anomaly (difference between annual SST and the long-term average) were obtained from the Australian Bureau of Meteorology at monthly intervals. From the Bureau of Meteorology, we also obtained monthly mean minimum air temperature (i.e. monthly mean of daily minimum) and monthly mean maximum air temperature (i.e. monthly mean of daily maximum) for the coastal station nearest to the centre of each latitudinal block. SST data from the wave-monitoring buoy network along the Queensland east coast were provided at 30-minute intervals by the Queensland Department of Science, Information Technology, Innovation and the Arts (DSITIA), under the Creative Commons Attribution 3.0 (CC BY) licence. Only the buoy (Datawell Waverider Buoy) located off North Stradbroke Island in the Moreton Bay region (27°29.285'S; 153° 37.931'E) had a time series with sufficient SST data for the purposes of the current analysis (January 1997 to December 2012).

Monthly maximum and mean freshwater discharge (m^3^s^−1^) data were downloaded from the Department of Natural Resources and Mines (http://watermonitoring.derm.qld.gov.au/host.htm [accessed June 30, 2013]), under the CC BY licence. Discharge data for the downstream gauging station of each drainage basin (33 in total) were binned to 1° latitudinal blocks, depending on where the point of seawards discharge was situated. Three monthly metrics of freshwater discharge were then calculated for each of the 13 latitudinal blocks: (1) peak discharge, which was the maximum rate of discharge across each latitudinal block in a given month; (2) mean discharge, which was monthly mean discharge averaged over each latitudinal block; and (3) cumulative mean discharge, which was the sum of monthly mean discharge across each latitudinal block.

Because marine mammals are influenced by both the past and present environment, predictor variables were lagged. Annual predictors (mean SST anomaly and mean SOI) were lagged at 1-yearly intervals to 5 years, i.e. a lag of 1 represented the previous year's environmental data tested against the present year's marine mammal mortality data. Monthly environmental predictors were lagged using moving window functions to examine both chronic and acute effects of environmental forcing in detail. Freshwater discharge was lagged at intervals of 0, 1, 3, 6, 9, 12 and 15 months (where a lag of 0 equalled the focal month for the response variable, and a lag of 1 equalled both the focal month and the previous month). *Lagged peak discharge* was calculated using a moving maximum function as the maximum rate of discharge over the time period. *Lagged cumulative discharge* was calculated using a moving cumulative function as the total cumulative mean discharge over the time period. *Lagged mean discharge* was calculated using a simple moving average (SMA) as the mean discharge over the time period. Air temperature was expected to affect marine mammals over shorter timescales than freshwater discharge and was therefore lagged at intervals of 0, 1, 2, 3, 4, 5 and 6 months using SMA functions. Cyclones were too infrequent to be lagged over shorter time periods and can have severe impacts on marine habitats that can take years to recover [29], [30]. The number of cyclones crossing each latitude block was therefore lagged using moving cumulative functions at intervals of 9, 12, 15, 18, 21, 24 and 30 months. SST was lagged using a SMA function at intervals of 0, 1, 2, 3, 4, 5, 6, 7, 9 and 12 months.

### Data analysis

Dugongs, as the only marine mammal herbivore, were analysed separately. Cetaceans were assigned to functional ecological groups, because individual species were too infrequent for detailed analysis. ‘Inshore dolphins’ included the three species of dolphins known to be resident in coastal waters: the ‘inshore’ Indo-Pacific bottlenose (*Tursiops aduncus*), Indo-Pacific humpback (*Sousa chinensis*) and Australian snubfin (*Orcaella heinsohni*) dolphins. ‘Offshore/migratory cetaceans’ included oceanic or neritic dolphins such as the ‘offshore’ bottlenose (*Tursiops truncatus*), short-beaked common (*Delphinus delphis*) and spinner (*Stenella longirostris*) dolphins, in addition to migratory species such as the humpback whale (*Megaptera novaeangliae*) and species occasionally present in coastal waters (e.g. pygmy sperm whale, *Kogia breviceps* and melon-headed whale, *Peponocephala electra*). Bottlenose dolphins not identified to species were not analysed because they could have been in either the inshore or the offshore groups.

We first examined the relationship between annual natural mortality and environmental predictors (SST anomaly and SOI) across the entire region. Environmental predictors were lagged at intervals of 1 to 5 years. Response variables were normalised using log transformation (log_e_ *x* + 1) and relationships were assessed using linear regression and standard regression diagnostics.

We then examined the full region-by-month dataset with the modelling approach recommended by Zuur *et al.* [31]. Briefly, temporal autocorrelation plots, spatial variograms, residual diagnostics and Akaike's Information Criterion (AIC) of different mixed models were used to determine the optimal variance structure. This indicated significant seasonal autocorrelation (Figures S1–S3), and that the optimal variance structure included separate intercepts for each region and year. In effect, this approach accounted for heterogeneity in the marine mammal mortality-environmental relationship between regions and years. A cubic-spline function of month (coded from 1 to 12) was used to model seasonal autocorrelation [32]. This general mixed-model structure was used to compare the effects of different environmental predictors on marine mammal mortality. Because there was no *a priori* expectation of a linear relationship between marine mammal mortality and environmental predictors, a generalised additive mixed modelling (GAMM) approach was used (library gamm4 v 0.1-6 of R v 3.0.1, the R foundation for statistical computing). Dugong monthly mortality data were log-normally distributed and were hence normalised by log*_e_*(*x*+1) transformation and analysed using a Gaussian error distribution. Inshore dolphin and offshore/migratory cetacean mortalities were modelled with a Poisson error distribution and a log-link function.

We analysed each set of lagged predictor variables separately by comparing the AIC of each model against the null model (i.e. the model including no environmental predictors). The best predictor variables were chosen based on (a) lowest AIC and (b) residual diagnostics. The final model was then chosen by comparing the AIC of different interactive and main effect models including the best predictor variables. Final models, including more than one predictor variable, were screened for collinearity (i.e. |r| < 0.4).

We then examined three marine mammal ‘hotspots’ separately (Townsville, Moreton Bay and Hervey Bay) using area-specific generalised additive models (GAM). The same general model structure for seasonal effects was used as above, but no random effects were included. The influence of SST was examined only for the Moreton Bay region between January 1997 and December 2012.

## Results

### Annual mortality and environmental forcing

**Figure 1:**
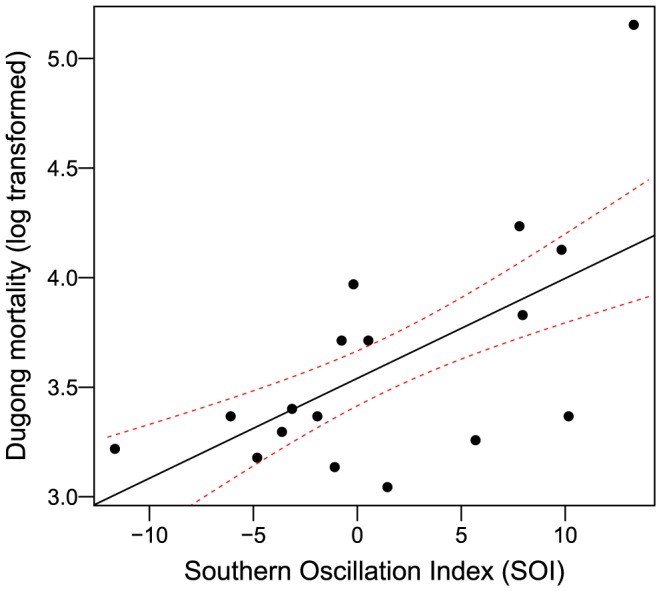
Relationship between annual natural mortality of dugongs and ENSO (SOI, annual average). The model was fit using robust regression (dotted red lines: 90% confidence intervals) and the outlier at the top of the graph is from 2011.

Annual dugong mortalities were significantly associated with SOI (b  =  0.052 ± 0.015; t  =  3.35; r^2^  =  0.417; df =  15; *p*-value  =  0.0044). To account for the clear outlier of 2011 (Figure 1), the regression was refit with this value excluded (b  =  0.0315 ± 0.013; t  =  2.34; r^2^  =  0.281; df =  14; *p*-value  =  0.035) and using robust regression (b  =  0.0456 ± 0.016; Library MASS, Huber's M-estimator; Figure 1). The relationship between annual dugong mortalities and SOI was not as strong for lagged SOI, remaining significant for a 1-year lag (b  =  0.048 ± 0.016; t  =  2.98; r^2^  =  0.372; df =  15; *p*-value  =  0.009) but not for lags of 2, 3, 4 and 5 years (*p*-values from 0.14 to 0.83). There was no significant relationship between annual dugong mortalities and SST temperature anomaly, either for the same year for lags up to 5 years (*p*-values from 0.13 to 0.95).

**Figure 2:**
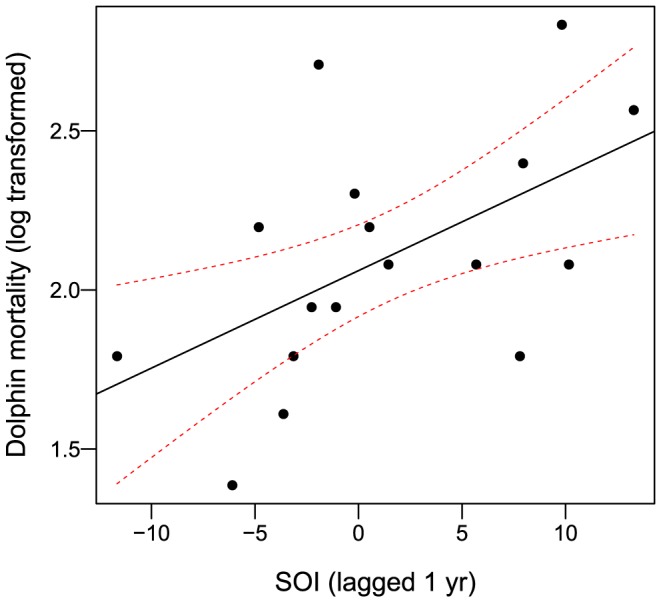
Relationship between annual natural mortality of inshore dolphins and ENSO (annual average SOI lagged by 1 year) (OLS regression; dotted red lines: 90% confidence intervals).

Annual mortality of inshore dolphins was significantly associated with SOI lagged by 1 year (b  =  0.030 ± 0.012; t  =  2.50; r^2^  =  0.29; df =  15; *p*-value  =  0.025, Figure 2), but not for the same year or at lags of 2, 3, 4 and 5 years (*p*-values from 0.11 to 0.94). Offshore/migratory cetacean mortality was not significantly associated with SOI (*p*-values from to 0.12 to 0.96) for lags of up to 5 years. SST anomaly influenced neither annual inshore dolphin mortality (*p*-values from 0.08 to 0.92, lagged up to 5 years) nor annual mortality of offshore/migratory cetaceans (*p*-values from 0.08 to 0.92, lagged up to 5 years).

### Monthly mortality and environmental forcing

**Figure 3:**
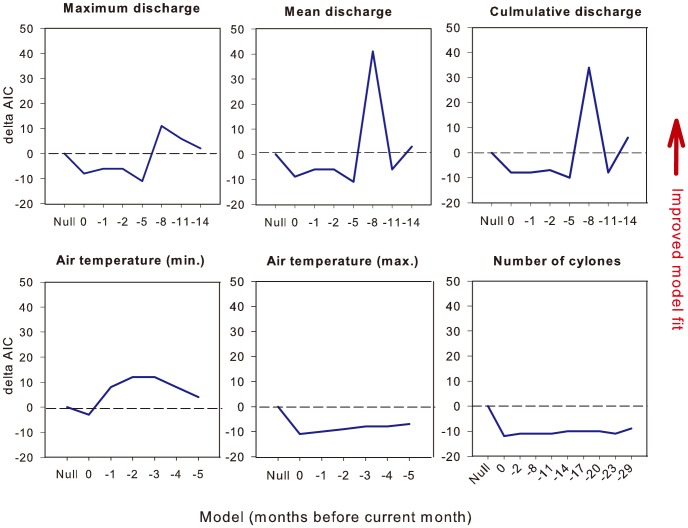
Fit of different additive models of dugong natural mortality as a function of lagged environmental predictors, at lags from zero (same month) up to 29 months (-29). In each case predictors are compared to the null model, which includes only the random effects and time-series component. Delta AIC represents the difference in predictive power between the null model and the model in question (higher values represent improved model fit).

The predictive power of GAMMs for monthly dugong mortality by region was not improved by including the number of cyclones crossing the coast or maximum air temperature (Figure 3). However, model fit was improved by including minimum air temperature lagged 2 or 3 months (i.e. by a 3- to 4-month SMA window including the focal month). A strong improvement in the predictive power of models occurred with the inclusion of mean discharge lagged by 8 months. To a lesser extent, cumulative discharge and maximum discharge also improved model fit for the same lagged time period.

**Figure 4:**
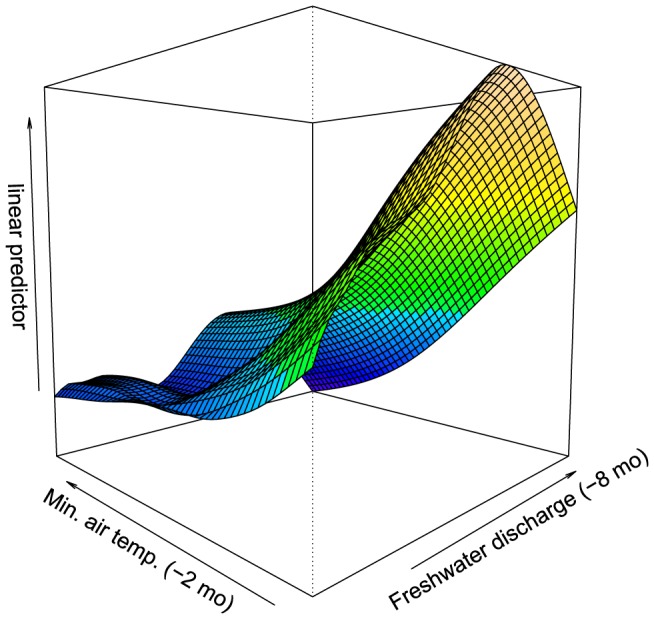
Final model of dugong natural mortality (linear predictor, log*_e_* x + 1) in response to mean minimum air temperature (°C, lagged by 2 months) and freshwater discharge (m^3^s^−1^, lagged by 8 months and square-root transformed).

On the basis of lowest AIC and residual diagnostics, the final model included the main effects of minimum air temperature (lagged by 2 months) and mean discharge (lagged by 8 months), in addition to an interaction between lagged mean discharge and lagged minimum air temperature (Figure 4). Freshwater discharge was square-root transformed to reduce the influence of outliers (i.e. major floods in large catchments). This model predicted that on average, i.e. across latitudinal regions, elevated dugong mortalities occur after a 9-month period of elevated rainfall and a 3-month period of low air temperatures. The interaction accounted for the fact that elevated dugong mortality was more closely linked to sustained freshwater discharge when it also coincided with prolonged low air temperatures.

**Figure 5:**
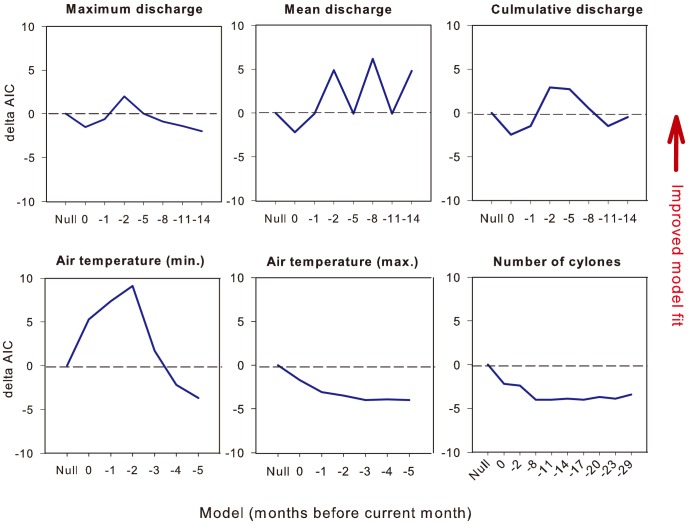
Fit of different additive models of inshore dolphin natural mortality as a function of environmental predictors at lags from zero (same month) up to 29 months (-29). Refer to the caption of Figure 3 for a full explanation.

**Figure 6:**
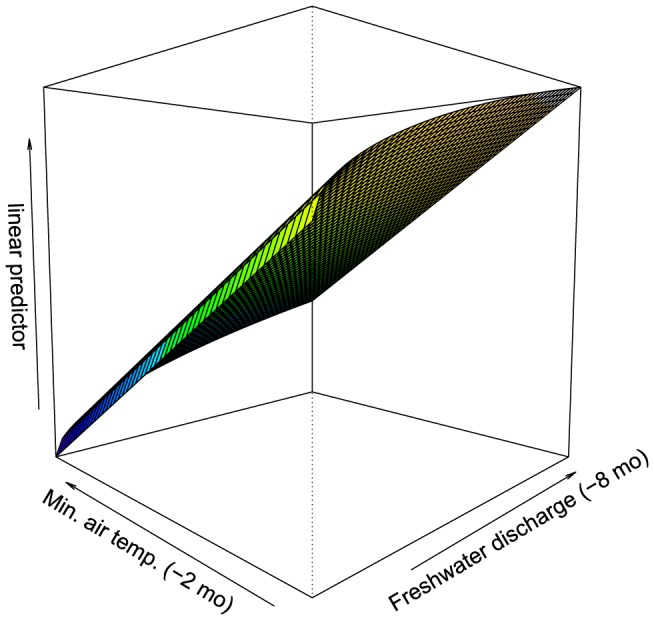
Final model of inshore dolphin natural mortality in response to mean minimum air temperature (°C, lagged by 2 months) and freshwater discharge (m^3^s^−1^, lagged by 8 months and square-root transformed). A Poisson error and log-link function were used for this relationship, the response surface is on the scale of the linear predictor.

A very similar pattern was evident for inshore dolphin mortality. Again, neither the number of cyclones nor maximum air temperature improved the fit of the GAMM (Figure 5). Of the predictors of freshwater discharge, lags of 2 months improved GAMM fit in each case, but the most pronounced improvement occurred when mean discharge was lagged by 8 months. Minimum air temperature lagged by 2 months also resulted in a marked improvement in the fit of the model. Using the same model selection criteria as above, we arrived at a final model that included the main effects of mean freshwater discharge (lagged by 8 months, square-root transformed) and minimum air temperature (lagged by 2 months) (Figure 6).

**Figure 7:**
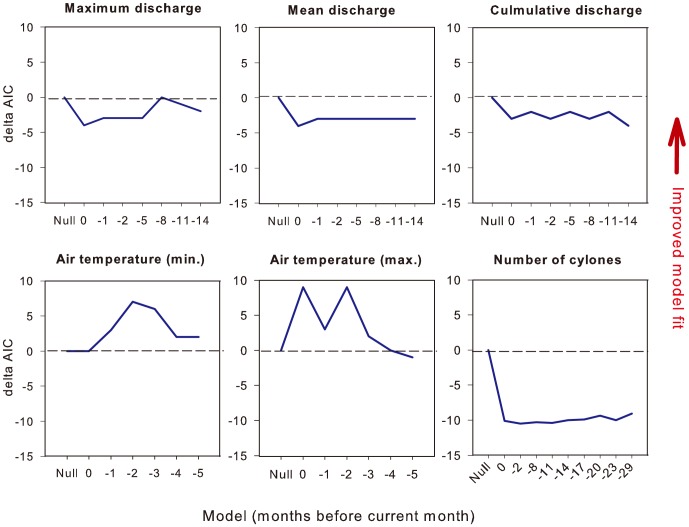
Fit of different additive models of offshore/migratory cetacean natural mortality as a function of environmental predictors at lags from zero (same month) up to 29 months (-29). Refer to the caption of Figure 3 for a full explanation.

**Figure 8:**
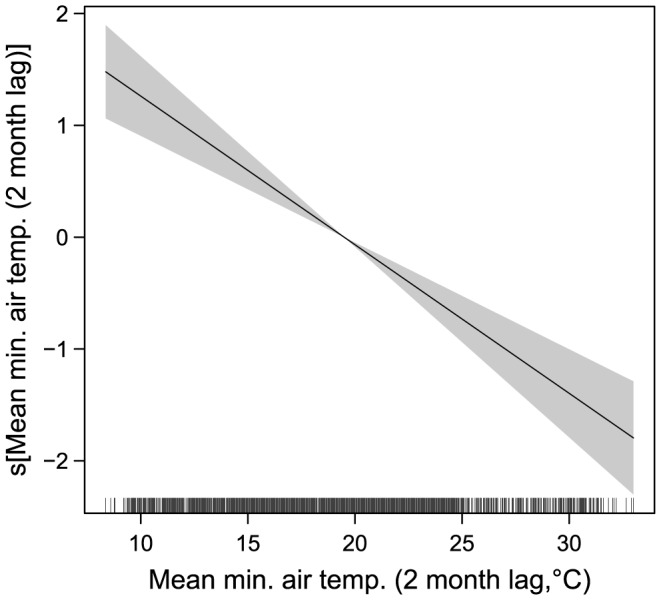
Relationship between offshore/migratory cetacean mortality and mean minimum air temperature (lagged by 2 months). The solid line is the smoother from the final GAMM model and the shaded area represents the 95% confidence intervals. The vertical black lines on the *x* axis denote individual values. A Poisson error and log-link function were used for this relationship, the response surface is on the scale of the linear predictor.

In contrast, freshwater discharge did not improve the fit of GAMMs for offshore/migratory cetaceans (Figure 7). This was also true for the number of cyclones, but minimum air temperature again improved model fit. As for dugongs and inshore dolphins, minimum air temperature lagged by 2 months improved model fit. However, in the case of offshore cetaceans, maximum air temperature (lagged by 2 months) also improved model fit. Examination of model residuals indicated that the maximum air temperature models were strongly influenced by outliers (i.e. extreme temperatures). We therefore arrived at a final model that included only the linear effect of lagged minimum air temperature (Figure 8).

### Area specific models: dugongs

For the Moreton Bay region, a model including the main effects of minimum air temperature (lagged by 2 months) and mean discharge (lagged by 8 months) best predicted dugong mortality. SST had a similar effect on dugong mortality as minimum air temperature (lagged by 2 months). A GAM including SST explained approximately 19.6% of deviance compared to 19.7% explained by the GAM including minimum air temperature (lagged by 2 months). There was significant co-variance between air temperature and SST, especially for mean minimum air temperature. Correlation between air temperature and SST was the strongest for data lagged by 2 months (r  =  0.955), as was also the case for maximum mean air temperature (r  =  0.926), suggesting that air temperature lagged by 2 months is a good proxy for SST in the region.

For the Hervey Bay region, the model with the lowest AIC scores was slightly different to the overall region-by-month mixed model, and included the main effects of cumulative discharge (lagged by 8 months) and maximum mean air temperature (lagged by 4 months). The model including only the main effects of cumulative discharge (lagged by 8 months, square-root transformed) and month, explained approximately 22.6% of deviance, whereas the model including only the main effect of maximum mean air temperature (lagged by 4 months) explained 23.9% of deviance. No SST data were available for the Hervey Bay region.

In the Townsville area, the final area-specific model included similar terms as the regional model, although cumulative discharge (lagged by 8 months, 42% of deviance, AIC = 240.86) performed better than mean discharge (lagged by 8 months, 41.9% of deviance, AIC = 306.95). A model including minimum air temperature (lagged by 2 months) explained 25.2% of deviance. The final model explained 39.5% of deviance and included cumulative discharge (lagged by 8 months, square root transformed), month and minimum air temperature (lagged by 2 months).

There were insufficient data to include the effects of cyclones for either the Moreton Bay or Hervey Bay regions. In the Townsville area, there were only enough data to fit a model for the cumulative number of cyclones lagged by 29 months, which explained 19.1% of deviance.

The area between Coolangatta and Hervey Bay was analysed separately for inshore dolphins, because the data did not support a detailed analysis for each individual area. Again, on the basis of AIC and deviance, the best predictors were lagged mean discharge (8 months, 22.9% of deviance) and lagged air temperature, although in this case minimum air temperature lagged by 1 month was the best fit (18.2% of deviance). The final model included minimum air temperature (lagged by 1 month) and mean discharge (lagged 8 months, square-root transformed) and explained 26.5% of deviance.

## Discussion

Freshwater discharge predicted mortality of inshore dolphins and dugongs in subtropical and tropical eastern Australia, and the relationship was the strongest for models that included lagged effects. The relationship was clear both across a very large spatial scale (>2000 km) and in empirical models specific to latitudinal regions, and underpinned correlations between inshore marine mammal mortality and SOI. As predicted, freshwater discharge was not an important driver of natural mortality in offshore or migratory cetaceans. To our knowledge, this is the first demonstration of a long-term relationship between freshwater discharge and mortality of inshore marine mammals in tropical or subtropical waters. Lagged air temperature also predicted mortality trends in dugongs, inshore dolphins and offshore/migratory marine mammals, although relationships were less consistent at a regional level.

Correlative spatio-temporal models are an important first step to understand and predict the timing and magnitude of mortality events. They are also a fundamental step towards generating testable hypotheses for the functional basis of observed relationships. To this end, the two most parsimonious functional explanations for the relationship between freshwater discharge and inshore dolphin and dugong mortality are (1) reduced food availability, and (2) direct impacts on health.

Strong support for the first hypothesis is available for dugongs. The density and species composition of seagrass pastures in inshore waters are closely related to freshwater discharge [33]. Elevated discharge reduces photosynthetic available radiation (PAR), exported sediment can smother seagrasses, and flood discharge can scour seagrass beds and seed banks [34]. Dugongs have a high metabolic demand and are thought to be impacted from food loss comparatively quickly [2]. The correlation between SOI (lagged by approximately 2 years) and nesting numbers of the only other large herbivorous marine species in the region, the green turtle, *Chelonia mydas*, also supports the contention that freshwater discharge can regulate populations of herbivores by limiting food availability [35], [36]. Reduced food availability is also the simplest explanation for the observed link between inshore dolphin mortality and freshwater discharge. Given that freshwater discharge has a strong influence on the distribution, abundance and phenology of many species of fish and crustaceans in eastern Australia (e.g. [23], [37] and other references herein), it would not be surprising to see these effects realised in apex predators.

The impact of sustained periods of elevated freshwater discharge (i.e. lagged mean discharge) on inshore marine mammals in our study was more pronounced than the effect of the magnitude of an individual flood event *per se* (i.e. lagged maximum discharge). Seagrass pastures of dugongs are known to be able to survive short-term periods of reduced PAR, but not sustained periods [38]. Similarly, while prey species of dolphins may be able to tolerate pulses of elevated discharge by moving to areas of higher salinity, sustained periods of rainfall can impose constraints on prey resources by limiting the availability of suitable habitat or by inhibiting post-larval recruitment [25], [39], [40].

Freshwater discharge may also impact on the health of marine mammals by increasing exposure to infectious pathogens such as toxoplasmosis and faecal coliform bacteria [41]–[43], or to contaminants with immunosuppressive effects [44]. Toxoplasmosis or toxoplasmosis antibodies have been observed in Indo-Pacific humpback dolphins [41] and dugongs [45] in Queensland. Elevated contaminant concentrations have also been reported in dugongs and inshore dolphins in the region [46]–[49], including levels of polychlorinated biphenyls where immunosuppression may occur based on dose-response relationships for other species [49].

Temperature also influences the health and physiology of marine mammals [50], [51], in addition to impacting foraging pasture [52] and prey production [53]. In our study, sustained low air temperature was associated with mortality of dugongs, inshore dolphins and offshore/migratory cetaceans. The very close correspondence between air temperature and SST in the Moreton Bay region suggests that lagged air temperature may simply be a good proxy of SST, rather than an important driver of marine mammal mortality in its own right.

There are several lines of evidence to suggest that the health of dugongs is limited by cooler temperatures at the southern limits of their range. First, seasonal movements are correlated to temperature changes [2], [54]–[56]. Second, dugongs seem to move further southwards along the east coast of Australia in warm years [57]. Finally, cold-stress syndrome has been described in dugongs in Moreton Bay [51]. Much less is known of the effects of temperature on tropical dolphins-most studies on the effects of temperature on mortality of cetaceans have focused on impacts in temperate regions [58], [59]. Elsewhere in the subtropics, sustained cold temperature was thought to be a contributing factor to the high mortality rate of bottlenose dolphins (*T. truncatus*) observed in the northern Gulf of Mexico after the Deepwater Horizon oil spill [43].

The fact that lagged minimum air temperature also explained mortality of dugongs in the tropics (i.e. Townsville region), where temperature is not expected to be cold enough to impair health, suggests that impacts on health may not be the only reason for the association between low temperature and mortality in our study. Other seasonal dynamics, such as the seasonal availability of seagrass pastures or prey, may have explained some of this association [60]. In the case of offshore/migratory cetaceans, the relationship between mortality and lagged air temperatures may be indicative of larger-scale climatic or oceanographic drivers, as the results of other recent studies imply [61], [62].

Despite the fact that cyclones are known to cause major disturbance to coastal ecosystems [24], [29], the number of cyclones crossing the coast did not improve the fit of the overall model, and only improved the fit of the Townsville regional model for dugong mortality. The main reason for this is that cyclones very rarely cross the Queensland coast in subtropical and southern areas, which meant that there were insufficient data for rigorous statistical analysis in the overall model.

The accuracy of predictive models of marine mammal mortality would be undoubtedly improved by including covariates such as population abundance. However, estimates of population abundance are still too inaccurate at a relevant spatial scale for this to be of value in our study area, even in the case of relatively well monitored species such as dugongs [63]. Even so, our study highlights the importance of incorporating past environmental forcing in developing predictive models for inshore marine mammal mortality, because the effects of temperature and freshwater discharge on inshore marine mammal mortality were not fully realised until they were lagged using moving average functions of 3 and 9 months, respectively.

Overall, our study provides an important basis for further investigation into the proximate basis of the functional relationship between marine mammal mortality and environmental forcing, ideally through detailed necropsies and dedicated surveys of population health. Further, our results illustrate how data collected from a marine mammal strandings network can be used in conjunction with routinely measured environmental and climatic variables to predict and prepare for periods of increased natural marine mammal mortality. Our study also highlights the need for further investigation into the influence of long-term trends of climate variability on marine mammals in tropical and subtropical regions. Current climate models do not have a strong degree of certainty in predicting how climate change will affect ENSO-related precipitation variability [64]. Yet, our results suggest that this aspect of climate may have a central role in the population dynamics of marine mammals in tropical-subtropical eastern Australia.

## Supporting Information

Figure S1 **Autocorrelation function (ASF) of dugong mortality.** Lags are in years and mortalities were summed across regions. Values outside the dotted lines are significant (α  =  0.05).
(EPS)

Figure S2 **Autocorrelation function (ASF) of inshore dolphin mortality.** Lags are in years and mortalities were summed across regions. Values outside the dotted lines are significant (α  =  0.05).
(EPS)

Figure S3 **Autocorrelation function (ASF) of offshore/migratory cetacean mortality.** Lags are in years and mortalities were summed across regions. Values outside the dotted lines are significant (α  =  0.05).
(EPS)

Figure S4 **Monthly dugong natural mortalities and environmental predictors (mean monthly freshwater discharge, lagged by 8 months; and mean minimum air temperature, lagged by 2 months) in the Moreton Bay region (27°S latitudinal block).**
(EPS)

Figure S5 **Monthly dugong natural mortalities and environmental predictors (mean monthly freshwater discharge, lagged by 8 months; and mean minimum air temperature, lagged by 2 months) in the Hervey Bay region (25°S latitudinal block).**
(EPS)

Figure S6 **Monthly dugong natural mortalities and environmental predictors (mean monthly freshwater discharge, lagged by 8 months; and mean minimum air temperature, lagged by 2 months) in the Townsville region (19°S latitudinal block).**
(EPS)

Figure S7 **Inshore dolphin natural mortalities and environmental predictors (mean monthly freshwater discharge, lagged by 8 months; and mean minimum air temperature, lagged by 2 months) in southern Queensland (24 to 28°S).**
(EPS)
